# Functional Neuroimaging in the New Era of Big Data

**DOI:** 10.1016/j.gpb.2018.11.005

**Published:** 2019-12-04

**Authors:** Xiang Li, Ning Guo, Quanzheng Li

**Affiliations:** Massachusetts General Hospital and Harvard Medical School, Boston, MA 02114, USA

**Keywords:** Big data, Neuroimaging, Machine learning, Health informatics, fMRI

## Abstract

The field of functional **neuroimaging** has substantially advanced as a **big data** science in the past decade, thanks to international collaborative projects and community efforts. Here we conducted a literature review on functional neuroimaging, with focus on three general challenges in big data tasks: data collection and sharing, data infrastructure construction, and data analysis methods. The review covers a wide range of literature types including perspectives, database descriptions, methodology developments, and technical details. We show how each of the challenges was proposed and addressed, and how these solutions formed the three core foundations for the functional neuroimaging as a big data science and helped to build the current data-rich and data-driven community. Furthermore, based on our review of recent literature on the upcoming challenges and opportunities toward future scientific discoveries, we envisioned that the functional neuroimaging community needs to advance from the current foundations to better data integration infrastructure, methodology development toward improved learning capability, and multi-discipline translational research framework for this new era of big data.

## Introduction

Functional neuroimaging techniques, including functional magnetic resonance imaging (fMRI) acquired both during task (tfMRI) and resting-state (rsfMRI), electroencephalography (EEG), magnetoencephalography (MEG), and other modalities, provide powerful tools to study human brain and serve as methodological foundations for quantative cognitive neuroscience [1]. Neuroimaging analysis of all these modalities has a long history of close relationship with data science, statistics, and medical imaging community. Similar to data analysis tasks in other applications, earlier work of functional neuroimaging analysis usually involved a very limited number of subjects. For example, the resting-state network study in [2] used the datasets that only consisted of 10 subjects in two separate scans to infer the networks, which was a similar case in [3] (10 subjects in two experiments) for studying functional connectivity in tfMRI. Decomposition of multi-subject fMRI analysis in [4] used 5 datasets, with each dataset consisting of 3 subjects as the model input. As more functional neuroimaging data become available and consequently more data-driven analytical approaches are adopted, in the past few decades, there has been growing awareness of the need and significance for a big data perspective on the neuroscience research. A series of literature discussed the new aims and characteristics of general and functional neuroimaging, both prospectively and retrospectively. Roberts et al. proposed that the brain functional fluctuation signal (both fMRI and EEG) follows non-Gaussian distribution and is intrinsically “heavy-tailed” [5], which is a common characteristic (“endogeneity”) of big data analytics [6], thus precluding the use of simple statistical models to fully capture the data distribution. In the special issue “Dealing with Data” of *Science*, Akil et al. [7] addressed the need for neuroinformatics system (as a “prelude to new discoveries”) to support the analytics on neuroimaging big data and enable coordination across multi-centered efforts under the Neuroscience Information Framework (NIF). They also proposed the concept of both macroscopic (*e.g.*, MRI and behavioral data) and microscopic (function and structure of individual neuron cells) connectomes to achieve the goal of deciphering the “neural choreography” of the brain at multiple scales spatiotemporally. From the machine learning perspective under a big data premise, traditional methods for functional neuroimaging analysis, which have been proven successful in multiple applications including functional network analysis and functional dynamics modeling [8], are in need of adaptation [9] to handle growing amount of big data for both computational capacity and speed [10] (*e.g.*, via parallel computing [11]). Later in the perspective article of [12] and [13], it was proposed that neuroimaging was an emerging example of discovery-oriented science based on big data, thus facing a broad range of challenges and risks which were commonly present in the field of data science, that is, challenges in handling the growing amount of the data, challenges in sharing and archiving the data, and challenges in exploring and mining the data. Coincidentally, these challenges and the corresponding solutions were also discussed in the perspective article on big data in the microscale connectomics in neuroscience [14], calling for better computation, better data sharing, and better data management. In the later sections we will review the community perspectives and efforts on addressing these three challenges.

## Better data availability

In the past decades we have seen a rapid growth in both the quantity and quality of the neuroimaging data on various scales, thanks to a series of collaborative projects on brain science with huge amount of functional neuroimaging data collected [15] (also refer to [16] for an extended review of neuroimaging projects), including the European Human Brain Project (reconstructing the brain’s multi-scale organization) [17], the U.S. Brain Activity Map (establishing functional connectome of the entire brain), the Allen Institute for Brain Science (developing cellular, gene, and structural atlas of brain) and the U.S. Brain Research through Advancing Innovative Neurotechnologies (BRAIN) Initiative (investigating the interaction among individual cells and complex neural circuits in the brain) [18]. As the data volume and number of investigation sites grow, challenges in social and logistic aspects in data sharing rise [19]. As pointed out in [20] as an analogue to the Human Genome Project, although the increasing quantity of neuroimaging data can help us to better model the brain function and structure, difficulty in data sharing, including the issues mentioned above and ethical concerns, motivations, and cultural shifts [21], can be more complex than expected. To address these challenges, a series of data sharing projects were established over the past decades, which provided values to the community including enhancing reproducibility, improving research value, and reducing research cost [22]. One of the data collection and neuroinformatics projects mentioned in [7], the ongoing Human Connectome Project (HCP) [23], collected neuroimaging (functional and structural MRI, EEG, and MEG) and behavioral data from 1200 subjects, with the data freely available to the community. The milestone paper initiating the 1000 Functional Connectomes Project (1000FCP) in [24] demonstrated the feasibility of collecting imaging data from a large number of subjects (1414 volunteers at 35 international centers) and analyzing them to establish a universal functional architecture to identify putative boundaries between functional networks in the brain. The subsequent project, the International Neuroimaging Data-sharing Initiative (FCP/INDI) [25], provided open access to imaging datasets of over thousands of subjects, both clinically and non-clinically. The Laboratory of Neuro Imaging (LONI) pipeline not only provided data access to nearly 1000 subjects specifically with a large population of Alzheimer’s disease (AD) [26], but also established an integrated data center for big data mining and knowledge discovery [27]. The Neuroimaging Informatics Tools and Resources Clearinghouse (NITRC) image repository [28] hosted 14 projects with neuroimaging data from 6845 subjects. Later development of data collection projects resulted in a substantial number of publicly-available neuroimaging databases, consisting of populations with schizophrenia [29], [30], attention deficit/hyperactivity disorder (ADHD) [31], autism spectrum disorder (ASD) [32], [33], development [34], mild cognitive impairment (MCI), AD [35], and aging [36], [37], as well as healthy controls for various research purposes [38], [39], [40], [41], [42]. The ongoing effort from UK Biobank study [43] aims to acquire multi-modal imaging data from 100,000 participants within various age groups, with currently 5000 participants' data being released. A summary of current prominent large databases that are publicly available is listed in Table 1. While most of the databases feature rsfMRI data (mainly for the purpose of connectivity analysis), some of them also include scans of tfMRI with task designs for specific purposes [44] (*e.g.*, memory and motor controls). In addition to these large data sharing efforts, which usually consist of healthy individuals and people with common diseases, more heterogeneous small datasets produced by individual groups are equally important to the community [45].

**Table 1:** **List of current publicly available functional neuroimaging databases**




*Note*: MRI, magnetic resonance imaging; fMRI: functional MRI; tfMRI, task-based functional MRI; rsfMRI: resting-state functional MRI; DW-MRI, diffusion-weighted MRI; EEG, electroencephalography; MEG, magnetoencephalography; PET, positron emission tomography; AD, Alzheimer's disease; MCI, mild cognitive impairment; EMCI, early MCI; LMCI, late MCI; ASD, autism spectrum disorder; ADHD, attention-deficit/hyperactivity disorder; SZ, schizophrenia.

It should also be noted that along with the growing size (*i.e.*, more subjects) of new functional neuroimaging databases, there have also been community efforts to improve the quality of the images acquired, which include but not limited to: (1) higher image resolution (both spatially and temporally) to support more precise localization and temporal characterization of brain function; (2) more imaging modalities; (3) more imaging sessions; and (4) specific target populations and/or specific cognition processes to identify new functional biomarkers [46]. These efforts are highly valuable to the community, especially in the emerging field of precision psychiatry [47].

## Better informatics infrastructure

Informatics infrastructure is a fundamental element to support large-scale data sharing and analytical work in neuroimaging big data. It is expected to enhance “*data collection, analysis, integration, interpretation, modeling, and dissemination*” as well as easier and more secure data sharing/collaboration [48]. Further requirements of the informatics infrastructure include management facility for better long-term data integrity, distributed computing and storage, as well as services for statistical computation, visualization, and interaction of the neuroimaging data [49]. The main archive and dissemination platform of HCP [23], the ConnectomeDB database [50], implements extensive automated validation for data quality control (QC) with manual examination of the data following standard operating procedures (SOPs), and allows multiple approaches for access (browser-base, hard drive, and Amazon S3). Both ConnectomeDB and NITRC utilize the infrastructure of the Extensible Neuroimaging Archive Toolkit (XNAT) software platform proposed in [51]. The platform provides functionality of data archiving, QC, and secure access for the neuroimaging data. It follows the three-tier architecture of (1) relational database, (2) Java-based middleware engine, and (3) browser-based user interface, implemented by XML schema. Based on the XNAT platform, multiple large-scale data sharing projects were developed. For example, the Northwestern University Neuroimaging Data Archive (NUNDA) [52] hosted 131 projects with 4783 subjects and 7972 imaging sessions. The Washington University Central Neuroimaging Data Archive (CNDA) [53] hosts more than 1000 research studies with 36,000 subjects and 60,000 image sessions. XNAT has then been further extended by the data dictionary service [54] to provide a flexible and multi-use data repository for the user. Various other neuroinformatics infrastructure were also developed to meet specific needs and scenarios, including the Data Processing & Analysis for Brain Imaging (DPABI) [55], which focused on test–retest reliability, QC, and streamlined preprocessing; the Holistic Atlases of Functional Networks and Interactomes (HAFNI)-enabled large-scale platform for neuroimaging informatics (HELPNI) [56], which specifically enables dictionary learning analysis on user-uploaded data based on XNAT; the work in [57], which can perform co-analysis between neuroimaging and genome-wide genotypes; and the BROCCOLI framework [58], which supports GPU acceleration.

## Better analytical methods

The early recognition for a data-driven (rather than model-driven), discovery-oriented neuroimaging analysis framework comes from [59], where the author envisioned that functional neuroimaging studies should merge the traditional, widely-used, hypothesis-based experiments with larger-scale discovery methods developed in data science fields. In the special issue “The Heavily Connected Brain” of *Science* [60], a similar argument was proposed on the neuroimage analytics: the over-simplified assumptions of the traditional study protocols need to be replaced by the unbiased, large-scale machine learning approaches to account for massive, dynamic interactions between brain regions. In the past decades, enormous machine learning techniques have been developed in the functional neuroimaging analysis community [8], for various purposes including classification, recognition, signal processing, and pattern discovery [61]. Methods for classifying abnormal brain functional patterns were developed for schizophrenia [62], AD [63], [64], MCI [65], [66], social anxiety [67], depression [68], [69], Parkinson's disease (PD) [70], post-traumatic stress disorder (PTSD) [71], ADHD [72], as well as other diseases [73]. Machine learning-based recognition/decoding of brain responses is another important task, analyzing brain processing such as language [74], visual stimuli of both low-level [75] and high-level context [76], emotion [77], and auditory [78]. These studies also constitute fundamentals to the development of brain-computer interface [79]. To discover brain functional patterns, especially its multi-level network structures, discovery models were applied to fully leverage the great details and depth of the current neuroimaging data [80], while various approaches have been further developed for characterizing dynamic network behavior of the brain [81], [82].

Shifting toward the big data perspective of functional neuroimaging on the basis of both higher image quality [83] and growing number of subjects [84], a series of modeling methods for large-scale functional neuroimaging data have been developed [85], mainly focused on unsupervised or semi-supervised matrix decomposition algorithms [86]. Earlier works in [87] proposed a data reduction scheme performing principle component analysis (PCA) before using independent component analysis (ICA) for group-wise functional network inference (GIFT toolbox). Similar scheme is later adopted by the extended GIFT [88] based on PCA compression and projection with various heuristics, such as only storing the lower triangular portion of covariance matrix [89]. Further, Rachakonda et al. [90] utilized subsampled eigenvalue decomposition techniques to approximate the subspace of the target decomposition, which can analyze group-wise PCA of 1600 subjects on a regular desktop computer. Other methods such as the group-wise PCA proposed in [91] can also achieve near-constant memory requirement for large datasets (>10,000 subjects) by iterative optimization. Dictionary learning-based methodologies for functional network inference have achieved similar performance. The HAFNI system [92], [93] found simultaneous, spatially-overlapping presence of both task-evoked and resting-state networks in the brain through sparse representation of whole-brain voxel-wise signals.

As the growth of neuroimaging dataset size quickly outnumbered the computational capacity of a single workstation, the need for parallel, computationally scalable framework becomes an important topic. The Thunder library [94] is an Apache Spark/MapReduced-based platform (https://spark.apache.org) for large-scale distributed computing of the neuroimaging data. Also based on Spark, distributed Singular Value Decomposition (SVD) [95] and low-rank matrix decomposition [96], [97] have been developed on large-scale functional neuroimaging data. Decentralized and cloud computing, which is a closely related methodology with parallel computing, has also been applied to neuroimaging analytics, including the decentralized data ICA [98] and Canadian Brain Imaging Research Platform (CBRAIN) [99].

**Figure 1:**
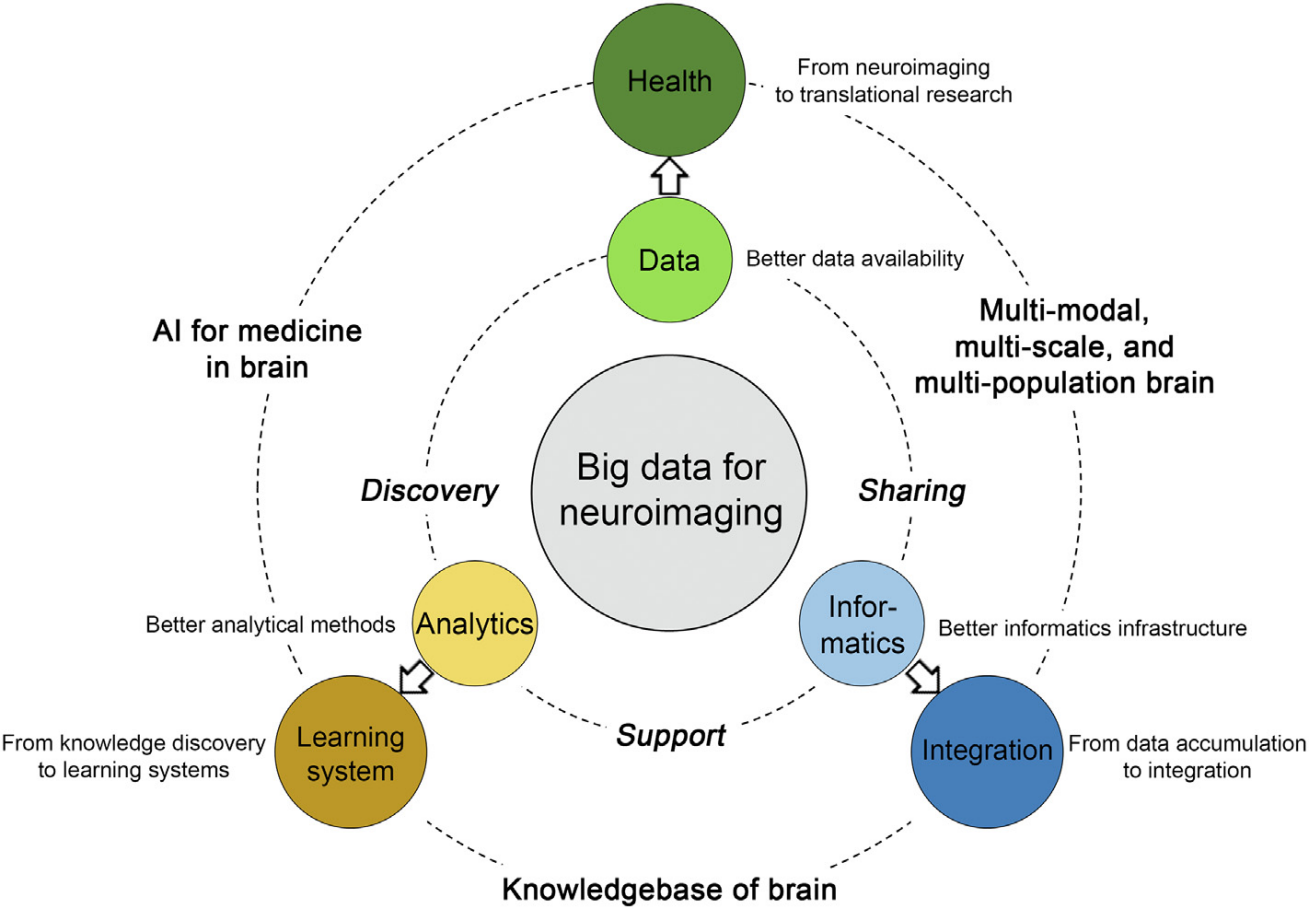
**Illustration of current and perspective core foundations of functional neuroimaging** Inner circle, three core foundations in big data for neuroimaging according to current literature. Major needs from the research community, knowledge discovery, computational support, and data sharing are fulfilled by them. Outer circle, perspectives on the advancement of these three foundations in the “new era of big data”, as well as the resulting new research focuses. Title of subsections in the manuscript are placed next to each core.

Led by the international projects and collaborative efforts, we have seen the advancement of functional neuroimaging as a big data science. At the same time, also in the past decade we have experienced a tremendous paradigm shift in the field of scientific research as a whole, toward data-intensive, discovery-oriented approaches, as envisioned in the book “The Fourth Paradigm” [100]. Data have been recognized not only as a valuable resource for research, but the research itself. Thus, we call the current stage of scientific research as in a “new era” of big data. The field of functional neuroimaging, after the efforts through all these years in amassing the data, building the infrastructures, and developing methodologies, begins the transformation as well as the new paradigm. Here in the later sections of this paper we review the literature focusing on potential advancement of the three core foundations (and challenges) discussed above, as illustrated in Figure 1.

## From neuroimaging to translational research

One major challenge of neuroimaging recently recognized by the field lies in its (relative) lack of impact on the clinical practice and public health [101], caused by limitations in both data and analytical methods [102]. The “translational neuroimaging” thus becomes an important goal for the field and has been addressed in multiple neuroscience research projects. For example, the China Brain Project specifically listed two translational aspects of brain science and neuroimaging in its framework: supporting better brain disease diagnosis and therapy and developing brain-machine intelligence [16]. In the BRAIN initiative, it was also proposed that a deeper understanding of human brain will bring corresponding clinical benefits, although not necessarily immediate [18]. Previous studies on computational methods for functional neuroimaging data (such as those based on network characterization) have shown potential capability in early diagnosis [103], intervention [104], and drug discovery [105], [106]. From a clinical perspective, as summarized in [107], functional neuroimaging in clinical practice has been proven useful for treatment planning and diagnostic supplement. Translational value of functional neuroimaging [108], especially its big data perspective, is specifically important for precision psychiatry [47], where it was addressed that availability of massively acquired data from multiple sources is the foundation for considering diverse patient characteristics, thus achieving “precision” during diagnosis and treatment. Also recognized in [47], in addition to imaging data, the solution to more practical clinical application is to introduce more biological data (“-omics”) into the analytics. Studies on imaging genomics have shown that the structure of subcortical brain regions can be altered by genetic variants [109], which might lead to neuropsychiatric dysfunction. Perspective work in [110] proposed the need for an integrated bioinformatics approach to correctly characterize bipolar disorder (BD) based on meta-analysis, addressing the insufficient predictive value from a single phenotype. It suggested utilizing multiple biomarkers from a series of personal profiling data to establish computational models, including neuroimaging, transcriptomics, proteomics, and metabolomics. On the investigation of other common psychiatric disorders including ASD and ADHD, it was proposed in [111] that in addition to MRI-based imaging features (*e.g.*, functional connectivity and gray matter volume), other imaging modalities such as EEG, MEG, or functional near-infrared spectroscopy (fNIRS) should also be considered. Ashar et al. [112] mapped imaging features to multi-output behavioral scores for AD and schizophrenia studies in a transfer learning manner, with the potential to combine heterogeneous data sources and imaging modalities. A recent review of studies on brain developmental disorders [113] shows that structural and functional imaging characteristics, combined with genetic and environmental influences, can provide early biomarkers with improved prediction power. Utilization of other data types, such as physiological and mechanistic data [114], can also help us reveal the underlying mechanisms of neuroimaging for its translational applications.

## From data accumulation to integration

While registration to common template has been a standard practice for MRI-based studies (which is the similar case for EEG), establishing a common ground for neuroimaging studies across different populations, scales, and imaging modalities is still a tremendous challenge. The Allen Brain Atlas showed a promising architecture for characterizing and localizing mouse brain on different scales, integrating MRI, serial two-photon (STP) tomography [115], and gene expression data [116] in a single atlas. For collaboration across institutions on neuroscience research, BigNeuron [117] proposed a new module of neuroimaging informatics to integrate the community efforts, in terms of both data and methodology, into a standard framework for the study of neural circuits and connectomics, especially on single-neuron morphology and reconstruction. On the human brain studies, the multi-modal parcellation framework proposed in [118] provided a way to obtain accurate areal map of the brain based on parcellation on cortical architecture, function, connectivity, and topography. Cortical parcellation framework only based on individual rsfMRI data but guided by population-level functional atlas was also proposed [119] and demonstrated similarly high level of reproducibility. Also based on rsfMRI, the “connectivity domain” framework proposed in [120] established a new common space to represent individual fMRI data, which has demonstrated advantages for the data-driven analysis. In addition, studies based on meta-analysis to integrate functional neuroimaging findings have been proposed to form a large-scale brain map [121].

## From knowledge discovery to learning systems

In the book that Goodfellow et al. laid the foundation of deep learning [122], the authors stated that the purpose of machine learning is to “discover not only the mapping from representation to output but also the representation itself”, which is known as representation learning. In other words, knowledge discovered from big data can also be massive and needs to be organized into practical models. The authors argued that learned representations, rather than the hand-crafted ones, can achieve better performance with greater robustness to new tasks. Such paradigm shift has also been recognized by medical and biological researchers. As proposed in [123], there is a need to transform the traditional “expert systems” which were pre-defined by domain knowledge, to the learning-based systems which can identify the most discriminative/predictive combinations (representation) from enormous number of factors. Similarly, perspective work in [124] proposed that advanced machine learning systems are desperately needed to leverage the vast volume of neuroimaging data. Capability of deep learning in exploiting hierarchical feature representations has shown superior performance in medical image analysis for the task of detection, classification, and prognosis [125]. One of the promising methodologies in deep learning for modeling the 4-D functional neuroimaging data is the recurrent neural network (RNN) [126] (see also [127] for its current implementation) based on long short-term memory (LSTM) [128], which utilizes cyclic connections to model sequences (*e.g.*, time frames). Various preliminary attempts have been made to apply RNN to functional neuroimaging data, including functional network identification [129], psychiatric disorder diagnosis [130], and estimation of long-term dependencies for the hemodynamic responses [131]. Other methods of applying deep learning-based methods to neuroimaging studies include the unsupervised autoencoder modeling of fMRI data [132], [133] and classical convolutional neural network [134].

## Concluding remarks

In this work, we reviewed studies and efforts on the advancement of functional neuroimaging as a big data science in the past decade. The field has been rapidly growing in data quantity, infrastructure, and analytical methods. At the same time, there are also growing concerns on the translational limitation of functional neuroimaging (*e.g.*, in clinical practice and drug discovery) and on the difficulty in fully leveraging the ever-growing amount of data. We then conducted focused review on the recent progress in both methodologies (specifically in deep learning) and research frameworks (in data integration and multi-type data collection) to address these concerns. We envision that in the new era of big data, the field of functional neuroimaging could be greatly enhanced by the tools and concepts in the new paradigm of scientific discovery, with promises of better understanding the brain functional architecture and more useful discoveries from the imaging data.

## Competing interests

The authors have declared no competing interests.

## References

[1] Glasser M.F., Smith S.M., Marcus D.S., Andersson J.L., Auerbach E.J., Behrens T.E. (2016). The Human Connectome Project's neuroimaging approach. Nat Neurosci.

[2] Damoiseaux J.S., Rombouts S.A., Barkhof F., Scheltens P., Stam C.J., Smith S.M. (2006). Consistent resting-state networks across healthy subjects. Proc Natl Acad Sci U S A.

[3] Fair D.A., Schlaggar B.L., Cohen A.L., Miezin F.M., Dosenbach N.U., Wenger K.K. (2007). A method for using blocked and event-related fMRI data to study “resting state” functional connectivity. NeuroImage.

[4] Beckmann C.F., Smith S.M. (2005). Tensorial extensions of independent component analysis for multisubject FMRI analysis. NeuroImage.

[5] Roberts J.A., Boonstra T.W., Breakspear M. (2015). The heavy tail of the human brain. Curr Opin Neurobiol.

[6] Fan J., Han F., Liu H. (2014). Challenges of Big Data analysis. Natl Sci Rev.

[7] Akil H., Martone M.E., Van Essen D.C. (2011). Challenges and opportunities in mining neuroscience data. Science.

[8] Lemm S., Blankertz B., Dickhaus T., Müller K.R. (2011). Introduction to machine learning for brain imaging. NeuroImage.

[9] Philip Chen C.L., Zhang C.Y. (2014). Data-intensive applications, challenges, techniques and technologies: a survey on Big Data. Inf Sci.

[10] Katal A, Wazid M, Goudar RH. Big data: issues, challenges, tools and good practices. 2013 6th Inter Conf Contemp Comput (IC3) 2013:404–9.

[11] Dean J., Ghemawat S. (2004). MapReduce: simplified data processing on large clusters. Commun ACM.

[12] Van Horn J.D., Toga A.W. (2014). Human neuroimaging as a “Big Data” science. Brain Imaging Behav.

[13] Smith S.M., Nichols T.E. (2018). Statistical challenges in “Big Data” human neuroimaging. Neuron.

[14] Lichtman J.W., Pfister H., Shavit N. (2014). The big data challenges of connectomics. Nat Neurosci.

[15] Kandel E.R., Markram H., Matthews P.M., Yuste R., Koch C. (2013). Neuroscience thinks big (and collaboratively). Nat Rev Neurosci.

[16] Grillner S., Ip N., Koch C., Koroshetz W., Okano H., Polachek M. (2016). Worldwide initiatives to advance brain research. Nat Neurosci.

[17] Amunts K., Ebell C., Muller J., Telefont M., Knoll A., Lippert T. (2016). The Human Brain Project: creating a European research infrastructure to decode the human brain. Neuron.

[18] Insel T.R., Landis S.C., Collins F.S. (2013). The NIH BRAIN Initiative. Science.

[19] Landhuis E. (2017). Neuroscience: big brain, big data. Nature.

[20] Choudhury S., Fishman J.R., McGowan M.L., Juengst E.T. (2014). Big data, open science and the brain: lessons learned from genomics. Front Hum Neurosci.

[21] Sejnowski T.J., Churchland P.S., Movshon J.A. (2014). Putting big data to good use in neuroscience. Nat Neurosci.

[22] Poldrack R.A., Gorgolewski K.J. (2014). Making big data open: data sharing in neuroimaging. Nat Neurosci.

[23] Van Essen D.C., Smith S.M., Barch D.M., Behrens T.E.J., Yacoub E., Ugurbil K. (2013). The WU-Minn Human Connectome Project: an overview. NeuroImage.

[24] Biswal B.B., Mennes M., Zuo X.N., Gohel S., Kelly C., Smith S.M. (2010). Toward discovery science of human brain function. Proc Natl Acad Sci U S A.

[25] Mennes M., Biswal B.B., Castellanos F.X., Milham M.P. (2013). Making data sharing work: the FCP/INDI experience. NeuroImage.

[26] Jack C.R., Bernstein M.A., Fox N.C., Thompson P., Alexander G., Harvey D. (2008). The Alzheimer's disease neuroimaging initiative (ADNI): MRI methods. J Magn Reson Imaging.

[27] Toga A.W., Foster I., Kesselman C., Madduri R., Chard K., Deutsch E.W. (2015). Big biomedical data as the key resource for discovery science. J Am Med Inform Assoc.

[28] Kennedy D.N., Haselgrove C., Riehl J., Preuss N., Buccigrossi R. (2016). The NITRC image repository. NeuroImage.

[29] Mayer A.R., Ruhl D., Merideth F., Ling J., Hanlon F., Bustillo J. (2013). Functional imaging of the hemodynamic sensory gating response in schizophrenia. Hum Brain Mapp.

[30] Keator D.B., van Erp T.G.M., Turner J.A., Glover G.H., Mueller B.A., Liu T.T. (2016). The Function biomedical informatics research network data repository. NeuroImage.

[31] Bellec P., Chu C., Chouinard-Decorte F., Benhajali Y., Margulies D.S., Craddock R.C. (2017). The neuro bureau ADHD-200 preprocessed repository. NeuroImage.

[32] Di Martino A., Yan C.G., Li Q., Denio E., Castellanos F.X., Alaerts K. (2013). The autism brain imaging data exchange: towards a large-scale evaluation of the intrinsic brain architecture in autism. Mol Psychiatry.

[33] Di Martino A., O’Connor D., Chen B., Alaerts K., Anderson J.S., Assaf M. (2017). Enhancing studies of the connectome in autism using the autism brain imaging data exchange II. Sci Data.

[34] Alexander L.M., Escalera J., Ai L., Andreotti C., Febre K., Mangone A. (2017). An open resource for transdiagnostic research in pediatric mental health and learning disorders. Sci Data.

[35] Rubin E.H., Storandt M., Miller J.P., Kinscherf D.A., Grant E.A., Morris J.C. (1998). A prospective study of cognitive function and onset of dementia in cognitively healthy elders. Arch Neurol.

[36] Kennedy K.M., Rodrigue K.M., Bischof G.N., Hebrank A.C., Reuter-Lorenz P.A., Park D.C. (2015). Age trajectories of functional activation under conditions of low and high processing demands: an adult lifespan fMRI study of the aging brain. NeuroImage.

[37] Wei D., Zhuang K., Chen Q., Yang W., Liu W., Wang K. (2018). Structural and functional MRI from a cross-sectional Southwest University Adult Lifespan Dataset (SALD). Sci Data.

[38] Zuo X.N., Anderson J.S., Bellec P., Birn R.M., Biswal B.B., Blautzik J. (2014). An open science resource for establishing reliability and reproducibility in functional connectomics. Sci Data.

[39] Liu W., Wei D., Chen Q., Yang W., Meng J., Wu G. (2017). Longitudinal test-retest neuroimaging data from healthy young adults in southwest China. Sci Data.

[40] Mendes N., Oligschlaeger S., Lauckner M.E., Golchert J., Huntenburg J.M., Falkiewicz M. (2019). A functional connectome phenotyping dataset including cognitive state and personality measures. Sci Data.

[41] Tian L., Wang J., Yan C., He Y. (2011). Hemisphere- and gender-related differences in small-world brain networks: a resting-state functional MRI study. NeuroImage.

[42] Nooner K., Colcombe S., Tobe R., Mennes M., Benedict M., Moreno A. (2012). The NKI-Rockland sample: a model for accelerating the pace of discovery science in psychiatry. Front Neurosci.

[43] Miller K.L., Alfaro-Almagro F., Bangerter N.K., Thomas D.L., Yacoub E., Xu J. (2016). Multimodal population brain imaging in the UK Biobank prospective epidemiological study. Nat Neurosci.

[44] Poldrack R.A., Barch D.M., Mitchell J.P., Wager T.D., Wagner A.D., Devlin J.T. (2013). Toward open sharing of task-based fMRI data: the OpenfMRI project. Front Neuroinform.

[45] Ferguson A.R., Nielson J.L., Cragin M.H., Bandrowski A.E., Martone M.E. (2014). Big data from small data: data-sharing in the “long tail” of neuroscience. Nat Neurosci.

[46] Tamminga C.A. (2014). Approaching human neuroscience for disease understanding. World Psychiatry.

[47] Fernandes B.S., Williams L.M., Steiner J., Leboyer M., Carvalho A.F., Berk M. (2017). The new field of ‘precision psychiatry’. BMC Med.

[48] Cheng X., Marcus D., Van Horn J.D., Luo Q., Mattay V.S., Weinberger D.R. (2015). Going beyond the current neuroinformatics infrastructure. Front Neuroinform.

[49] Freeman J. (2015). Open source tools for large-scale neuroscience. Curr Opin Neurobiol.

[50] Hodge M.R., Horton W., Brown T., Herrick R., Olsen T., Hileman M.E. (2016). ConnectomeDB—sharing human brain connectivity data. NeuroImage.

[51] Marcus D.S., Olsen T.R., Ramaratnam M., Buckner R.L. (2007). The extensible neuroimaging archive toolkit. Neuroinformatics.

[52] Alpert K., Kogan A., Parrish T., Marcus D., Wang L. (2016). The Northwestern University Neuroimaging Data Archive (NUNDA). NeuroImage.

[53] Gurney J., Olsen T., Flavin J., Ramaratnam M., Archie K., Ransford J. (2017). The Washington University Central Neuroimaging Data Archive. NeuroImage.

[54] Herrick R., McKay M., Olsen T., Horton W., Florida M., Moore C. (2014). Data dictionary services in XNAT and the Human Connectome Project. Front Neuroinform.

[55] Yan C.G., Wang X.D., Zuo X.N., Zang Y.F. (2016). DPABI: data processing & analysis for (resting-state) brain imaging. Neuroinformatics.

[56] Makkie M., Zhao S., Jiang X., Lv J., Zhao Y., Ge B. (2015). HAFNI-enabled largescale platform for neuroimaging informatics (HELPNI). Brain Inform.

[57] Da Mota B., Tudoran R., Costan A., Varoquaux G., Brasche G., Conrod P. (2014). Machine learning pattern for neuroimaging-genetic studies in the cloud. Front Neuroinform.

[58] Eklund A., Dufort P., Villani M., LaConte S. (2014). BROCCOLI: software for fast fMRI analysis on many-core CPUs and GPUs. Front Neuroinform.

[59] Smith S.M. (2012). The future of fMRI connectivity. NeuroImage.

[60] Turk-Browne N.B. (2013). Functional interactions as big data in the human brain. Science.

[61] Pereira F., Mitchell T., Botvinick M. (2009). Machine learning classifiers and fMRI: a tutorial overview. NeuroImage.

[62] Bleich-Cohen M., Jamshy S., Sharon H., Weizman R., Intrator N., Poyurovsky M. (2014). Machine learning fMRI classifier delineates subgroups of schizophrenia patients. Schizophr Res.

[63] Moradi E., Pepe A., Gaser C., Huttunen H., Tohka J. (2015). Alzheimer's Disease Neuroimaging Initiative. Machine learning framework for early MRI-based Alzheimer's conversion prediction in MCI subjects. NeuroImage.

[64] Suk H.I., Wee C.Y., Lee S.W., Shen D. (2016). State-space model with deep learning for functional dynamics estimation in resting-state fMRI. NeuroImage.

[65] Suk H.I., Lee S.W., Shen D. (2014). Alzheimer's Disease Neuroimaging Initiative. Hierarchical feature representation and multimodal fusion with deep learning for AD/MCI diagnosis. NeuroImage.

[66] Ou J., Xie L., Li X., Zhu D., Terry D., Puente A.N. (2015). Atomic connectomics signatures for characterization and differentiation of mild cognitive impairment. Brain Imaging Behav.

[67] Månsson K.N.T., Frick A., Boraxbekk C.J., Marquand A.F., Williams S.C.R., Carlbring P. (2015). Predicting long-term outcome of Internet-delivered cognitive behavior therapy for social anxiety disorder using fMRI and support vector machine learning. Transl Psychiatry.

[68] Zeng L.L., Shen H., Liu L., Wang L., Li B., Fang P. (2012). Identifying major depression using whole-brain functional connectivity: a multivariate pattern analysis. Brain.

[69] Wu M.J., Mwangi B., Bauer I.E., Passos I.C., Sanches M., Zunta-Soares G.B. (2017). Identification and individualized prediction of clinical phenotypes in bipolar disorders using neurocognitive data, neuroimaging scans and machine learning. NeuroImage.

[70] Abós A., Baggio H.C., Segura B., García-Díaz A.I., Compta Y., Martí M.J. (2017). Discriminating cognitive status in Parkinson’s disease through functional connectomics and machine learning. Sci Rep.

[71] Li X., Zhu D., Jiang X., Jin C., Zhang X., Guo L. (2014). Dynamic functional connectomics signatures for characterization and differentiation of PTSD patients. Hum Brain Mapp.

[72] Ou J., Lian Z., Xie L., Li X., Wang P., Hao Y. (2014). Atomic dynamic functional interaction patterns for characterization of ADHD. Hum Brain Mapp.

[73] Huys Q.J.M., Maia T.V., Frank M.J. (2016). Computational psychiatry as a bridge from neuroscience to clinical applications. Nat Neurosci.

[74] Mitchell T.M., Shinkareva S.V., Carlson A., Chang K.M., Malave V.L., Mason R.A. (2008). Predicting human brain activity associated with the meanings of nouns. Science.

[75] Kamitani Y., Tong F. (2005). Decoding the visual and subjective contents of the human brain. Nat Neurosci.

[76] Kay K.N., Naselaris T., Prenger R.J., Gallant J.L. (2008). Identifying natural images from human brain activity. Nature.

[77] Wang X.W., Nie D., Lu B.L. (2014). Emotional state classification from EEG data using machine learning approach. Neurocomputing.

[78] Formisano E., De Martino F., Valente G. (2008). Multivariate analysis of fMRI time series: classification and regression of brain responses using machine learning. Magn Reson Imaging.

[79] Vidaurre C., Sannelli C., Müller K.R., Blankertz B. (2011). Machine-learning-based coadaptive calibration for brain-computer interfaces. Neural Comput.

[80] Mišić B., Sporns O. (2016). From regions to connections and networks: new bridges between brain and behavior. Curr Opin Neurobiol.

[81] Preti M.G., Bolton T.A., Van De Ville D. (2017). The dynamic functional connectome: state-of-the-art and perspectives. NeuroImage.

[82] Yuan J., Li X., Zhang J., Luo L., Dong Q., Lv J. (2018). Spatio-temporal modeling of connectome-scale brain network interactions via time-evolving graphs. NeuroImage.

[83] Coalson T.S., Van Essen D.C., Glasser M.F. (2018). The impact of traditional neuroimaging methods on the spatial localization of cortical areas. Proc Natl Acad Sci U S A.

[84] Calhoun V.D., Liu J., Adalı T. (2009). A review of group ICA for fMRI data and ICA for joint inference of imaging, genetic, and ERP data. NeuroImage.

[85] Williams N., Henson R.N. (2018). Recent advances in functional neuroimaging analysis for cognitive neuroscience. Brain Neurosci Adv.

[86] Cichocki A., Mandic D., Lathauwer L.D., Zhou G., Zhao Q., Caiafa C. (2015). Tensor decompositions for signal processing applications: from two-way to multiway component analysis. IEEE Signal Process Mag.

[87] Calhoun V.D., Adali T., Pearlson G.D., Pekar J.J. (2001). A method for making group inferences from functional MRI data using independent component analysis. Hum Brain Mapp.

[88] Erhardt E.B., Rachakonda S., Bedrick E., Allen E., Adali T., Calhoun V.D. (2011). Comparison of multi-subject ICA methods for analysis of fMRI data. Hum Brain Mapp.

[89] Calhoun V.D., Silva R.F., Adalı T., Rachakonda S. (2015). Comparison of PCA approaches for very large group ICA. NeuroImage.

[90] Rachakonda S., Silva R.F., Liu J., Calhoun V.D. (2016). Memory efficient PCA methods for large group ICA. Front Neurosci.

[91] Smith S.M., Hyvärinen A., Varoquaux G., Miller K.L., Beckmann C.F. (2014). Group-PCA for very large fMRI datasets. NeuroImage.

[92] Lv J., Jiang X., Li X., Zhu D., Zhang S., Zhao S. (2015). Holistic atlases of functional networks and interactions reveal reciprocal organizational architecture of cortical function. IEEE Trans Biomed Eng.

[93] Lv J., Jiang X., Li X., Zhu D., Chen H., Zhang T. (2015). Sparse representation of whole-brain fMRI signals for identification of functional networks. Med Image Anal.

[94] Freeman J., Vladimirov N., Kawashima T., Mu Y., Sofroniew N.J., Bennett D.V. (2014). Mapping brain activity at scale with cluster computing. Nat Methods.

[95] Boubela R.N., Kalcher K., Huf W., Nasel C., Moser E. (2016). Big data approaches for the analysis of large-scale fMRI data using Apache Spark and GPU processing: a demonstration on resting-state fMRI data from the Human Connectome Project. Front Neurosci.

[96] Li X., Makkie M., Lin B., Fazli M.S., Davidson I., Ye J. (2016). Scalable fast rank-1 dictionary learning for fMRI big data analysis. Proceedings of the 22nd ACM SIGKDD International Conference on Knowledge Discovery and Data Mining.

[97] Makkie M., Li X., Quinn S., Lin B., Ye J., Mon G. (2019). A distributed computing platform for fMRI big data analytics. IEEE Trans Big Data.

[98] Baker BT, Silva RF, Calhoun VD, Sarwate AD, Plis SM. Large scale collaboration with autonomy: decentralized data ICA. 2015 IEEE 25th Inter Workshop Mach Learn Signal Process (MLSP) 2015:1–6.

[99] Sherif T., Rioux P., Rousseau M.E., Kassis N., Beck N., Adalat R. (2014). CBRAIN: a web-based, distributed computing platform for collaborative neuroimaging research. Front Neuroinform.

[100] Hey T., Tansley S., Tolle K. (2009). The fourth paradigm: data-intensive scientific discovery.

[101] Woo C.W., Chang L.J., Lindquist M.A., Wager T.D. (2017). Building better biomarkers: brain models in translational neuroimaging. Nat Neurosci.

[102] Schleim S., Roiser J.P. (2009). fMRI in translation: the challenges facing real-world applications. Front Hum Neurosci.

[103] Stephan K.E., Iglesias S., Heinzle J., Diaconescu A.O. (2015). Translational perspectives for computational neuroimaging. Neuron.

[104] Bakker A., Albert M.S., Krauss G., Speck C.L., Gallagher M. (2015). Response of the medial temporal lobe network in amnestic mild cognitive impairment to therapeutic intervention assessed by fMRI and memory task performance. NeuroImage: Clin.

[105] Wise R.G., Tracey I. (2006). The role of fMRI in drug discovery. J Magn Reson Imaging.

[106] Borsook D., Becerra L., Hargreaves R. (2006). A role for fMRI in optimizing CNS drug development. Nat Rev Drug Discov..

[107] Khanna N., Altmeyer W., Zhuo J., Steven A. (2015). Functional neuroimaging: fundamental principles and clinical applications. Neuroradiol J.

[108] Matthews P.M., Honey G.D., Bullmore E.T. (2006). Applications of fMRI in translational medicine and clinical practice. Nat Rev Neurosci.

[109] Hibar D.P., Stein J.L., Renteria M.E., Arias-Vasquez A., Desrivières S., Jahanshad N. (2015). Common genetic variants influence human subcortical brain structures. Nature.

[110] McIntyre R.S., Cha D.S., Jerrell J.M., Swardfager W., Kim R.D., Costa L.G. (2014). Advancing biomarker research: utilizing ‘Big Data’ approaches for the characterization and prevention of bipolar disorder. Bipolar Disord.

[111] Uddin L.Q., Dajani D.R., Voorhies W., Bednarz H., Kana R.K. (2017). Progress and roadblocks in the search for brain-based biomarkers of autism and attention-deficit/hyperactivity disorder. Transl Psychiatry.

[112] Ashar Y.K., Andrews-Hanna J.R., Dimidjian S., Wager T.D. (2017). Empathic care and distress: predictive brain markers and dissociable brain systems. Neuron.

[113] Gilmore J.H., Knickmeyer R.C., Gao W. (2018). Imaging structural and functional brain development in early childhood. Nat Rev Neurosci.

[114] Fox A.S., Shackman A.J. (2019). The central extended amygdala in fear and anxiety: closing the gap between mechanistic and neuroimaging research. Neurosci Lett.

[115] Oh S.W., Harris J.A., Ng L., Winslow B., Cain N., Mihalas S. (2014). A mesoscale connectome of the mouse brain. Nature.

[116] Lein E.S., Hawrylycz M.J., Ao N., Ayres M., Bensinger A., Bernard A. (2007). Genome-wide atlas of gene expression in the adult mouse brain. Nature.

[117] Peng H., Hawrylycz M., Roskams J., Hill S., Spruston N., Meijering E. (2015). BigNeuron: large-scale 3D neuron reconstruction from optical microscopy images. Neuron.

[118] Glasser M.F., Coalson T.S., Robinson E.C., Hacker C.D., Harwell J., Yacoub E. (2016). A multi-modal parcellation of human cerebral cortex. Nature.

[119] Wang D., Buckner R.L., Fox M.D., Holt D.J., Holmes A.J., Stoecklein S. (2015). Parcellating cortical functional networks in individuals. Nat Neurosci.

[120] Iraji A., Calhoun V.D., Wiseman N.M., Davoodi-Bojd E., Avanaki M.R.N., Haacke E.M. (2016). The connectivity domain: analyzing resting state fMRI data using feature-based data-driven and model-based methods. NeuroImage.

[121] Yarkoni T., Poldrack R.A., Nichols T.E., Van Essen D.C., Wager T.D. (2011). Large-scale automated synthesis of human functional neuroimaging data. Nat Methods.

[122] Goodfellow I., Bengio Y., Courville A. (2016). Deep Learning.

[123] Obermeyer Z., Emanuel E.J. (2016). Predicting the future-big data, machine learning, and clinical medicine. N Engl J Med.

[124] Vu M.A.T., Adalı T., Ba D., Buzsáki G., Carlson D., Heller K. (2018). A shared vision for machine learning in neuroscience. J Neurosci.

[125] Shen D., Wu G., Suk H.I. (2016). Deep learning in medical image analysis. Annu Rev Biomed Eng.

[126] Bengio Y., Simard P., Frasconi P. (1994). Learning long-term dependencies with gradient descent is difficult. IEEE Trans Neural Netw.

[127] Gers F.A., Schraudolph N.N., Schmidhuber J. (2003). Learning precise timing with lstm recurrent networks. J Mach Learn Res.

[128] Hochreiter S., Schmidhuber J. (1997). Long short-term memory. Neural Comput.

[129] Cui Y., Zhao S., Chen Y., Han J., Guo L., Xie L. (2019). Modeling brain diverse and complex hemodynamic response patterns via deep recurrent autoencoder. IEEE Trans Cogn Develop Syst.

[130] Durstewitz D., Koppe G., Meyer-Lindenberg A. (2019). Deep neural networks in psychiatry. Mol Psychiatry.

[131] Güçlü U., van Gerven M.A.J. (2017). Modeling the dynamics of human brain activity with recurrent neural networks. Front Comput Neurosci.

[132] Huang H., Hu X., Zhao Y., Makkie M., Dong Q., Zhao S. (2018). Modeling task fMRI data via deep convolutional autoencoder. IEEE Trans Med Imaging.

[133] Zhao Y., Dong Q., Chen H., Iraji A., Li Y., Makkie M. (2017). Constructing fine-granularity functional brain network atlases via deep convolutional autoencoder. Med Image Anal.

[134] Zhao Y., Li X., Zhang W., Zhao S., Makkie M., Zhang M. (2018). Modeling 4D fMRI data via Spatio-Temporal Convolutional Neural Networks (ST-CNN). Inter Conf Med Image Comput Comput Assist Interv (MICCAI).

